# Area-level factors associated with variation in involuntary psychiatric hospitalisation across England: a cross-sectional, ecological study

**DOI:** 10.1007/s00127-024-02748-5

**Published:** 2024-08-27

**Authors:** Matilda Minchin, Colette Christiansen, Lucy Maconick, Sonia Johnson

**Affiliations:** 1 Division of Psychiatry, University College London, Maple House, 149 Tottenham Court Road, London, W1T 7BN UK; 2 The Open University, Walton Hall, Milton Keynes, MK7 6AA UK; 3 Camden and Islington NHS Foundation Trust, St Pancras Hospital, 4 St Pancras Way, London, NW1 0PE UK

**Keywords:** Involuntary treatment, Mental health act, Mental health, Ecological, England

## Abstract

**Purpose:**

Involuntary hospitalisations for mental health care are rising in many high income countries, including England. Looking at variation between areas can help us understand why rates are rising and how this might be reversed. This cross-sectional, ecological study aimed to better understand variation in involuntary hospitalisations across England.

**Method:**

The unit of analysis was Clinical Commissioning Groups (CCGs), NHS bodies responsible for delivering healthcare to local areas in England. 205 CCGs were included in the analysis. Demographic, clinical, and socioeconomic variables at CCG-level were extracted from national, open access data bases. The outcome variable was the rate of involuntary hospitalisation for psychiatric care under the 1983 Mental Health Act in 2021/22.

**Results:**

There was a four-fold difference between the CCGs with the highest and lowest involuntary hospitalisations. In an adjusted analysis, CCGs with a higher percentage of severe mental illness in the population, higher percentage of male population, and higher community and outpatient mental health care use showed a higher rate of involuntary hospitalisation. Depression, urbanicity, deprivation, ethnicity, and age were not strongly associated with involuntary hospitalisation after adjustment. These variables explained 10.68% of the variation in involuntary hospitalisations across CCGs.

**Conclusion:**

Some demographic and clinical variables explained variation in involuntary hospitalisation between areas in England, however, most of the variance was unexplained. Complex relationships between urbanicity, deprivation, age, and ethnicity need to be further explored. The role of other influences, such as variation in service organisation or clinical practice, also need to be better understood.

**Supplementary Information:**

The online version contains supplementary material available at 10.1007/s00127-024-02748-5.

## Introduction

Over the past 50 years, there has been a marked effort to transfer inpatient mental health care to community settings [1]. Despite this, the number of people being involuntarily hospitalised for mental health care is rising in many European countries [2]. England especially has seen large increases in involuntary detentions under the 1983 Mental Health Act (MHA) [2, 3]. Under the MHA, a person with a mental disorder can be treated in hospital without consent if approved professionals agree the person is a risk to themselves or others and treatment is necessary [4].

Involuntary hospitalisation often leads to longer hospital stays, higher readmission, and higher suicide rates than voluntary treatment [5, 6]. While involuntary hospitalisation can ensure access to necessary mental health care, many people describe the experience as confusing, frightening, and unhelpful [7]. It is therefore important to understand why rates of involuntary hospitalisation are rising. Currently, the most supported theories for this rise are changes in legislation, service provision, and demographic factors [2, 3, 8].

At the individual level, greater risk of involuntary hospitalisation has been linked to severe mental illness, male gender, single marital status, unemployment, unstable accommodation, and lower previous contact with mental health services [9–11]. However, many of these sociodemographic groups are not increasing substantially or universally across countries [3, 12, 13], and so cannot fully explain rising involuntary hospitalisations. A recent meta-analysis found that Black, Asian, and Minority Ethnic (BAME) and migrant groups, especially those from Black Caribbean and Black African backgrounds, have considerably higher rates of involuntary hospitalisation [14]. Rising numbers of people from BAME or migrant backgrounds in Europe could therefore explain some of the rise in MHA use [2, 3]. Some suggest increased involuntary hospitalisation of BAME and migrant groups may be due to lower contact with mental health services, higher perceived risk of violence, or higher police contact [10, 14]. However, others suggest that this increased involuntary hospitalisation can be explained by these groups having, on average, lower age, higher prevalence of mental illness, and living in more urban areas [15–17].

Area-level variation in involuntary hospitalisation may also help us understand why rates are rising and how we can counteract this rise. There is large variation in involuntary hospitalisation rates between countries, however, this variation is poorly explained by differences in population demographics or legal systems [2]. Complex relationships within countries, between demographics, service provision, and sociocultural context, may instead influence the rate of involuntary hospitalisation [2]. Within England, previous ecological studies have found higher MHA use in areas with higher deprivation, urbanicity, and ethnic diversity [18, 19]. Areas with better community services also saw higher involuntary hospitalisations, perhaps as these services better identify those in need of involuntary treatment [19]. However, Weich et al. [19] report that variation in MHA use across England was mostly unexplained by these factors. The authors note that a substantial proportion of this remaining variance could be explained by psychiatric variables, such as diagnoses and clinical status [19].

The current study aimed to assess whether area-level variables can explain variation in MHA use across healthcare provider areas (Clinical Commissioning Groups; CCGs) in England. This was done using 2021/22 MHA detention statistics from the Mental Health Services Data Set [20]. Unlike previous population studies conducted in England [18, 19], we included data on psychiatric diagnoses in the population, alongside other demographic, service-use, and socioeconomic variables. The study also used more recent MHA detention data to account for changes in demographics and government policies that have substantially influenced mental health care provision in England over the past 10 years [3, 8]. We aimed to further clarify causes of variation in MHA use across England and point to potential explanations for rising rates of involuntary hospitalisation.

## Methods

This study used a cross-sectional, ecological design to assess variation in involuntary hospitalisation under the MHA across CCGs in England in 2021/22. CCGs (replaced by Integrated Care Boards in 2022) were National Health Service (NHS) bodies responsible for planning and delivering healthcare across geographical areas covering 500,000 people on average [20, 21]. As of April 2021, there were 106 CCGs in England. CCGs were overseen by their respective NHS England regional area [22]. There were seven NHS England regional areas in 2021. Information on 2021 CCG and NHS England region boundaries was sourced from the Office for National Statistics [23]. CCGs aimed to place people detained under the MHA in local services, and usually fewer than 1% of adult mental health bed days were inappropriate out-of-area placements [24, 25]. As such, we expect the “spill-over effect” between CCGs to be small.

### Outcome and explanatory variables

The primary outcome was the rate of involuntary hospitalisation under the MHA in each CCG from the 1st of April 2021 to the 31st of March 2022. This was calculated using the raw count of MHA detentions and the base population of each CCG. MHA detentions per 100,000 population have been used in figures to ease interpretation. Data on MHA detentions and base populations for each CCG was extracted from the Mental Health Services Data Set, a national data set using secondary data from mental health service providers [20]. This data set includes anyone involuntarily admitted to hospital under the MHA, but not police use of Sect. 136 to transport people to a place of safety. All 106 CCGs had complete data for the outcome variable.

Explanatory variables for the analysis were chosen based on their availability for 2021 CCG boundaries and their evidenced effect on involuntary hospitalisation rates [11, 14, 19]. Included CCGs had complete data for all variables. A full description of the search strategy and the variables extracted can be found in the Supplementary Material (page 2). Table 1 describes the outcome and explanatory variables used in this study, their source, and the date they were extracted.

**Table 1:** Definitions and data sources of the outcome and explanatory variables used

Variable Used	Data Source	Date Extracted	Definition
**Outcome Variable**			
Detentions under the Mental Health Act 1983	NHS Digital Mental Health Services Data Set	2021/22	People subject to detention under the Mental Health Act expressed as a *rate* of the base population of each CCG. Available for all ages.
**Main Analysis**			
Severe Mental Illness	NHS Digital Quality and Outcomes Framework	2020/21	The number of patients on GP registers recorded as having schizophrenia, bipolar affective disorder, or other psychoses as a *percentage* of all people on GP registers. Available for all ages.
Depression	NHS Digital Quality and Outcomes Framework	2020/21	The number of patients on GP registers aged 18 + with a new diagnosis of depression recorded between April 1st 2020 – March 31st 2021 as a *percentage* of all people on GP registers.
Deprivation	PHE’s Fingertips Database (Indicator 93275)	2019	The Index of Multiple Deprivation Score of each CCG - an overall measure of multiple deprivation experienced by people living in an area. Available for all ages.
Non-White Ethnicity	PHE’s Fingertips Database (Indicator 93267)	2011	*Percentage* of population reporting to be Non-White UK (includes Gypsy, Roma, and Traveller and Northern Irish people). Available for all ages. Data from the 2011 Office for National Statistics Census.
Age	Office for National Statistics	2020 (Mid-2020 Population Estimates)	*Percentage* of the population who were aged 18 to 35. Data extrapolated from the 2011 Office for National Statistics Census.
Sex	Office for National Statistics	2020 (Mid-2020 Population Estimates)	*Percentage* of the population who record their sex as “male”. Available for all ages. Data extrapolated from the 2011 Office for National Statistics Census.
Community and outpatient mental health service visits	PHE’s Fingertips Database (Indicator 93622)	2019/20	Directly standardised rate of attended non-inpatient contacts with secondary mental health services, *per 100*,*000* population. Available for all ages.
Urbanicity	Office for National Statistics	2021	2011 Rural-Urban Classifications- “Predominantly Urban”, “Urban with Significant Rural”, and “Predominantly Rural”.

*Note* NHS: National Health Service; CCG: Clinical Commissioning Group; PHE: Public Health England; GP: General Practitioner

### Sensitivity analysis

Data on ethnicity was only available at CCG-level from the Office for National Statistics 2011 Census [26]. To provide a more current estimate, a sensitivity analysis was performed using ethnicity data from the 2021 Census, which was reported for Sub-Integrated Care Boards [27]. When CCGs were abolished in 2022, they were converted into Sub-Integrated Care Boards. These can be linked through identity codes [28].

### Statistical analysis

The main analysis assessed the association between eight explanatory variables and the rate of MHA detentions across 106 CCGs in England in 2021/22. NHS Dorset CCG was excluded as they recorded a very low rate of MHA detentions in 2021/22 (3.90 per 100,000, 2.98 SDs from the mean), leaving 105 CCGs for the final analysis. We attributed this low value to measurement bias as NHS Dorset CCG has reported very few MHA detentions since Dorset Healthcare University NHS Foundation Trust stopped providing MHA information in 2019/20 [20]. Before this timepoint, NHS Dorset CCG reported around 80 involuntary hospitalisations per 100,000 population [20].

Due to overdispersion in the outcome variable, negative binomial regressions were used. Model selection was based on the Akaike Information Criterion and the Bayesian Information Criterion, with lower scores indicating a model with better fit while penalising for complexity [29]. Both fixed-effects and mixed-effects models, with an added random effect of NHS England region, were tested for goodness-of-fit. The random effect of NHS England region was added as CCGs grouped in the same region may share service-level characteristics. The full model selection process is outlined in the Supplementary Material (page 3). To understand the variance in MHA detentions across CCGs explained by the study variables, fixed-effects models were run and the Pseudo R^2^ was reported [30].

Firstly, univariate negative binomial regressions were conducted between each explanatory variable and the rate of MHA detentions. Explanatory variables that were significant to *p* < .05 were added to a final multivariate negative binomial model. This process was repeated for the sensitivity analysis. To avoid over-weighting of variables with larger scales, the number of community and outpatient mental health visits was transformed from per 100,000 to per 100 population.

We conducted a subgroup analysis based on urbanicity, as evidence suggests that urban and rural areas may show different risk factors for MHA detention [18]. To create more even groups, CCGs classified as “Predominantly Rural” and “Urban with Significant Rural” were considered rural, while CCGs classified as “Predominantly Urban” were considered urban. Data was analysed using Stata 17 software [31].

## Results

**Fig. 1:**
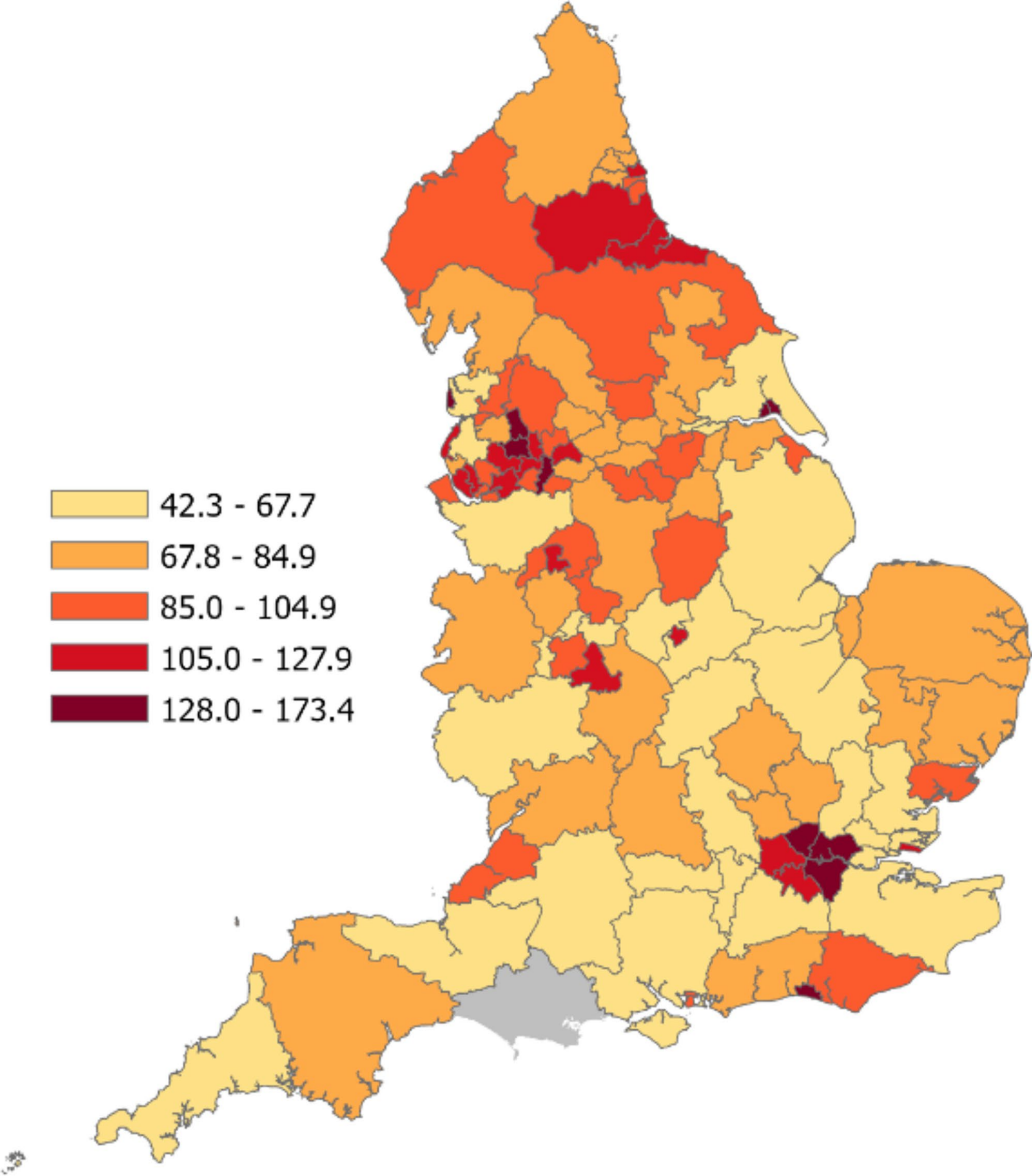
Mental Health Act Detentions (2021/22) per 100,000 Population Across Clinical Commissioning Groups in England. *Note* NHS Dorset CCG is grey due to being excluded from the study

**Fig. 2:**
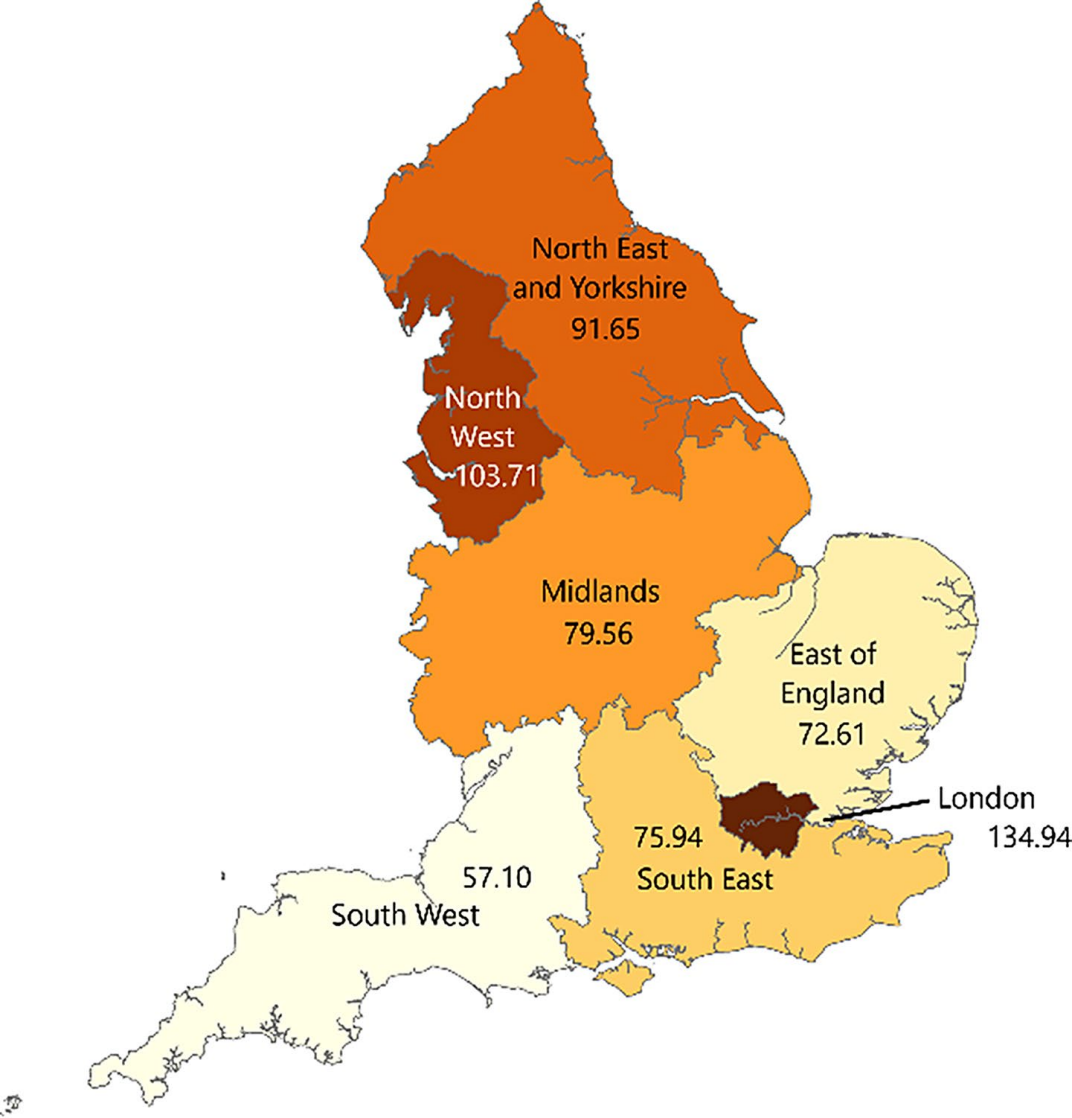
Mental Health Act Detentions (2021/22) per 100,000 Population Across NHS England Regions

105 CCGs, clustered into seven NHS England regions, were analysed to assess variation in use of the MHA. The rate of MHA detentions per 100,000 population in 2021/22 ranged from 42.30 in NHS Surrey Heartlands CCG to 173.40 in NHS Blackpool CCG, with an average of 88.97 per 100,000 (SD = 27.13). Within NHS England regions, mean MHA detentions per 100,000 ranged from 65.97 in NHS England South West to 134.94 in NHS England London. Maps showing MHA detentions per 100,000 population across CCGs and NHS England regions can be seen in Figs. 1 and 2, respectively. Table 2 describes the descriptive characteristics of the 105 included CCGs.

**Table 2:** Descriptive characteristics of the included 105 clinical commissioning groups in England

Main Analysis Variables	Mean (SD)	Range
MHA Detentions per 100,000	88.97 (27.13)	42.30 to 173.40
% Severe Mental Illness	0.93 (0.17)	0.61 to 1.55
% Depression	13.24 (2.31)	7.26 to 19.79
Index of Multiple Deprivation	22.98 (7.90)	10.03 to 45.04
% Non-White	10.33 (10.55)	1.45 to 49.48
% Aged 18 to 35 Years	22.35 (4.10)	16.17 to 38.38
% Male Sex	49.37 (0.63)	47.55 to 51.13
Community and Outpatient Mental Health Visits per 100	31.01 (8.25)	16.55 to 57.18
**Urbanicity**	**N**	**%**
Predominantly Rural	19	18.10
Urban with Significant Rural	20	19.05
Predominantly Urban	66	62.86

*Note* MHA : Mental Health Act

### Variables associated with MHA detentions

Univariate mixed-effects analyses were run between each explanatory variable and the rate of MHA detentions in 2021/22 across CCGs in England. CCGs with higher levels of severe mental illness (IRR = 3.226, 95% CI [2.452 to 4.245]), deprivation (IRR = 1.023, 95% CI [1.017 to 1.029]), non-White population (IRR = 1.011, 95% CI [1.006 to 1.016]), population aged 18 to 35 (IRR = 1.032, 95% CI [1.022 to 1.043]), male population (IRR = 1.172, 95% CI [1.092 to 1.258]), and community and outpatient mental health visits per 100 population (IRR = 1.019, 95% CI [1.013 to 1.025]) had higher rates of MHA detention to *p* < .001. Percentage of depression in the population was not associated with the rate of MHA detentions (IRR = 1.002, 95% CI [0.976 to 1.028], *p* = .896).

The rate of MHA detentions was lowest in areas rated “Urban with Significant Rural” (M = 68.89, SD = 15.65), higher in areas rated “Predominantly Rural” (M = 75.28, SD = 13.69), and highest in “Predominantly Urban” areas (M = 99.00, SD = 27.81). In a univariate mixed-effects analysis, areas that were rated as “Predominantly Urban” were associated with a higher rate of MHA detentions than those rated “Predominantly Rural” (IRR = 1.213, 95% CI [1.067 to 1.378], *p* = .003). Areas rated “Urban with Significant Rural” did not show a difference in MHA use compared to “Predominantly Rural” areas (IRR = 0.930, 95% CI [0.800 to 1.081], *p* = .342).

Severe mental illness, age, sex, ethnicity, deprivation, urbanicity, and community and outpatient service use were added to a multivariate mixed-effects model. In this model, the percentage of severe mental illness in the population was strongly associated with the rate of MHA detentions between CCGs (IRR = 2.327, 95% CI [1.803 to 3.003], *p* < .001). For each standard deviation increase in the percentage of severe mental illness in the population (SD = 0.17%), the rate of MHA detentions increased by 22.56%. The number of community and outpatient mental health visits per 100 population was also strongly associated with the rate of MHA detentions (IRR = 1.013, 95% CI [1.008 to 1.017], *p* < .001). For each standard deviation increase in community and outpatient mental health visits per 100 population (SD = 8.25), the rate of MHA detentions increased by 10.73%. The percentage of male population was moderately associated with the rate of MHA detentions (IRR = 1.085, 95% CI [1.015 to 1.160], *p* = .016), with a standard deviation increase in percentage of male population (SD = 0.63%) increasing the rate of MHA detentions by 5.36%. The percentage of non-White population was not strongly associated with the rate of MHA detentions (IRR = 1.003, 95% CI [0.999 to 1.007], *p* = .097). For a standard deviation increase in non-White population (SD = 10.55%), the rate of MHA detentions increased by 3.17%. No other variables were associated with MHA detentions in the multivariate mixed-effects model. Full results from the mixed-effects analyses can be seen in Table 3. The multivariate fixed-effects model produces a pseudo R^2^ of 0.1068 (see Supplementary Material, page 4). This suggests that the explanatory variables explain 10.68% of the variance in MHA detentions between CCGs. There were very few differences between the fixed-effects and mixed-effects models.

**Table 3:** Mixed-effects negative binomial regression for the rate of mental health act detention across clinical commissioning groups in England

	Univariate Analysis			Multivariate Analysis		
Variable	IRR	95% CI	*p* value	IRR	95% CI	*p* value
Severe Mental Illness	3.226	2.452 to 4.245	**< 0.001**	2.327	1.803 to 3.003	**< 0.001**
Depression	1.002	0.976 to 1.028	0.896	-	-	-
Index of Multiple Deprivation	1.023	1.017 to 1.029	**< 0.001**	1.000	0.994 to 1.007	0.883
% Non-White	1.011	1.006 to 1.016	**< 0.001**	1.003	0.999 to 1.007	0.097
% Aged 18 to 35 Years	1.032	1.022 to 1.043	**< 0.001**	1.001	0.990 to 1.013	0.820
% Male Sex	1.172	1.092 to 1.258	**< 0.001**	1.085	1.015 to 1.160	**0.016**
Community and Outpatient Mental Health Visits per 100	1.019	1.013 to 1.025	**< 0.001**	1.013	1.008 to 1.017	**< 0.001**
**Urbanicity**						
Predominantly Rural^†^	-	-	**-**	-	-	**-**
Urban with Significant Rural	0.930	0.800 to 1.081	0.342	0.962	0.870 to 1.065	0.445
Predominantly Urban	1.213	1.067 to 1.378	**0.003**	1.052	0.956 to 1.157	0.301

*Note* Bold indicates significance to *p* < .05

^†^ Reference category

### Urban and rural areas subgroup analysis

Two mixed-effects negative binomial regressions were conducted for CCGs classified as “Predominantly Urban”, considered urban, and those classified as “Predominantly Rural” and “Urban with Significant Rural”, considered rural. As seen in Table 4, the results are similar. Severe mental illness was a strong predictor of increased rate of MHA detentions in both rural (IRR = 2.473, 95% CI [1.431, 4.272] *p* = .001) and urban CCGs (IRR = 2.233, 95% CI [1.555, 3.207] *p* < .001). Higher percentage of male population was moderately associated with higher MHA detentions in urban CCGs (IRR = 1.072, 95% CI [0.996 to 1.154] *p* = .064) and rural CCGs (IRR = 1.170, 95% CI [1.008, 1.359] *p* = .039). Higher community and outpatient mental health visits were moderately associated with higher MHA detentions in rural CCGs (IRR = 1.009, 95% CI [1.001, 1.016] *p* = .027) and strongly associated in urban CCGs (IRR = 1.015, 95% CI [1.009, 1.021] *p* < .001). If a Bonferroni correction is adopted [32], meaning *p* < .025 to indicate significance for an original alpha level of 0.05, male sex is no longer significantly associated with MHA detention in both urban and rural areas. Furthermore, community and outpatient mental health visits are no longer associated with MHA use in rural areas. However, this is likely due to reduced power in the subgroup analysis [32]. No association was seen between the percentage of non-White population and MHA use in either rural or urban areas.

**Table 4:** Mixed-effects negative binomial regression for the rate of mental health act detention between urban and rural clinical commissioning groups

	Rural (*N* = 39)			Urban (*N* = 66)		
Variable	IRR	95% CI	*p* value	IRR	95% CI	*p* value
Severe Mental Illness	2.473	1.431 to 4.272	**0.001**	2.233	1.555 to 3.207	**< 0.001**
Deprivation	1.001	0.983 to 1.020	0.918	1.000	0.992 to 1.007	0.913
% Non-White	1.000	0.977 to 1.024	0.985	1.003	0.998 to 1.008	0.202
% Aged 18 to 35 Years	1.004	0.978 to 1.030	0.763	1.002	0.989 to 1.015	0.741
% Male Sex	1.170	1.008 to 1.359	**0.039**	1.072	0.996 to 1.154	0.064
Community and Outpatient Mental Health Visits per 100	1.009	1.001 to 1.016	**0.027**	1.015	1.009 to 1.021	**< 0.001**

*Note* Bold indicates significance to *p* < .05. Rural = Areas Classified as “Predominantly Rural” and “Urban with Significant Rural” under 2011 Rural-Urban Classification; Urban = Areas Classified as “Predominantly Urban” under 2011 Rural-Urban Classification.

### Sensitivity analysis

A sensitivity analysis was conducted for non-White ethnicity using the percentage of non-White population from the 2021 Census. The percentage of non-White population in CCGs in 2021 ranged from 3.92 to 73.06 (M = 19.85, SD = 14.99). The original and sensitivity non-White variables were highly correlated (*r* = .98). Similar to the original variable, the percentage of Non-White population in 2021 was associated with the rate of MHA detentions in the univariate mixed-effects analysis (IRR = 1.008, 95% CI [1.004 to 1.012], *p* < .001), but not strongly associated in the multivariate mixed-effects analysis (IRR = 1.002, 95% CI [0.999 to 1.005], *p* = .117). In the multivariate mixed-effects analysis, for each standard deviation increase in non-White population (SD = 14.99%) the rate of MHA detentions increases by 3.00%. All other results in the multivariate analysis were relatively unchanged (See Supplementary Material, page 5).

## Discussion

In line with previous research [2, 18, 19], we found large variation in involuntary hospitalisation under the MHA across areas in England. There was a four-fold difference between the CCGs with the highest and lowest rates. In an adjusted analysis, CCGs with a higher percentage of severe mental illness, higher male population, and more community and outpatient mental health visits showed higher MHA use. Urbanicity, ethnicity, age, and deprivation were not strongly associated with MHA use when analyses were adjusted.

Higher severe mental illness in the population was the factor most strongly associated with higher MHA use in the current study. The level of depression, however, was not associated with MHA use in unadjusted or adjusted analyses. This finding builds on previous ecological studies conducted in England, which have so far been unable to control for differences in levels of severe mental illness between areas [18, 19]. Studies of individuals have consistently linked severe mental illness to involuntary hospitalisation [9, 11, 33, 34], suggesting that involuntary mental health treatment is most used for those with bipolar or psychotic disorders. This is likely due to reduced capacity to consent and perceived higher risk [35], meaning people with these conditions more often meet the criteria for involuntary detention. However, it is unclear whether levels of severe mental illness are rising substantially [12, 36], and so this is unlikely to fully explain rising involuntary hospitalisations.

Areas with a higher proportion of males also showed higher involuntary hospitalisations in our study. Males may exhibit higher perceived or actual dangerousness, meaning they are seen as a higher risk to themselves or others, a key factor for MHA detention [37]. Higher detention of males may also relate to differences in help-seeking behaviour [38]. Males are less likely to be in contact with mental health services [39, 40], which may lead to more involuntary hospitalisations when experiencing a mental health crisis [11, 41]. While male gender and reduced contact with mental health services are seen to separately predict involuntary hospitalisation [11], these factors have not yet been linked empirically.

We found higher community and outpatient mental health use to be associated with higher involuntary hospitalisations across CCGs. This association remained after controlling for other area-level factors, including the percentage of severe mental illness. Previously, higher community healthcare spending and better community services have been linked to higher involuntary hospitalisations [2, 19, 42]. This is concerning as greater access to community treatment should reduce the need for involuntary inpatient care [8, 9, 43]. It could be the case that greater access to community treatment means those in need of involuntary hospitalisation are better identified [19]. However, as involuntary treatment often results in poor outcomes [5, 6], these findings suggest that community services need to be improved to provide earlier interventions for mental health and reduce unnecessary involuntary hospitalisation.

We did not find higher urbanicity, deprivation, or the percentage of population aged 18 to 35 to be associated with higher involuntary hospitalisation in the adjusted analysis. This is mostly in contradiction with previous work [15, 16, 18, 19], and could suggest that these factors do not affect involuntary hospitalisations when other variables are accounted for. Furthermore, an area having a higher percentage of non-White population was not strongly associated with higher MHA detentions in the adjusted analysis. Similar work has found that increased detention of BAME populations is mostly attributable to these populations showing higher severe mental illness, younger age, and living in urban areas [15, 16, 18]. However, higher urbanicity, non-White population, deprivation, and percentage of people aged 18 to 35 were all associated with higher MHA use in the unadjusted analyses. Complex relationships between these variables and mental illness may complicate our results [47, 48], masking each variable’s individual contribution at the area-level. For example, an area having higher deprivation, urbanicity, young population, and non-White population could contribute to higher SMI and, in turn, higher MHA use. Future research should aim to disentangle these relationships, ideally using longitudinal methods, to better understand how these variables interact to increase use of the MHA. As the population in England is rising, particularly in urban areas which show higher rates of ethnic diversity, deprivation, younger population, and mental illness [18], this could explain some of the rise in involuntary hospitalisation.

To explore these relationships further, we looked at urban and rural areas separately. However, we found no clear evidence that risk factors differ based on urbanicity. These findings differ from Keown et al. [18], who suggested that younger age and higher ethnic density were associated with involuntary hospitalisation in urban areas only. Our results suggest that when all factors are considered, higher area-level ethnic density and younger age profile are not risk factors for involuntary hospitalisation in either rural or urban areas. Our results may differ due to the addition of area-level severe mental illness in our study.

Explanatory variables used in the current study explained 10.68% of the variance in MHA detentions between CCGs. This is similar to Weich et al. [19] and suggests that, even with the addition of severe mental illness, there are further variables that produce variation in involuntary hospitalisation across England. We controlled for clustering within NHS England regions that oversaw CCGs, but the addition of these regions to the analysis did not improve the model fit. This suggests that little variance in MHA detention was explained by differences in service provision between NHS England regions. While NHS England regions oversaw CCGs, the roles of these groups were not always well defined, and mental health service provision may instead be more influenced by NHS Mental Health Trusts [49]. Differences in service provision and implementation of the MHA may explain some of the remaining variance between areas. Examining these differences further may highlight overuse or misuse of the MHA, which can be targeted to reduce involuntary hospitalisations. We were not able to include area-level indicators for migration, marital status, accommodation, employment, and substance misuse, which may also explain some of the remaining variance [9–11, 16].

### Strengths and limitations

The current study used national, open access data sets to better understand what causes variation in involuntary hospitalisation between areas in England. The completeness of the data and the addition of demographic, clinical, and socioeconomic variables is a strength. Unlike previous population studies conducted in England [18, 19], we included the percentage of mental illness in the population. Mental illness is an important factor identified in the literature [11], and its addition to the current study helps us understand what variables increase the rate of MHA detentions when controlling for area-level severe mental illness. Furthermore, by using recent data we have accounted for changes in demographics and government policy that have influenced involuntary hospitalisations in England over the past 10 years [3, 8].

One limitation of the current study is the quality of MHA data reported to the Mental Health Services Data Set [20]. The Mental Health Services Data Set quality report assesses the completeness and coverage of MHA data by comparing current data to older, but more complete, datasets [50]. They report that many eligible healthcare providers did not provide data on MHA use and others provided incomplete data. However, it is difficult to assess the extent of these issues across time and geographical regions due to changing CCG boundaries, organisations closing or merging, and differing methods across datasets. Dorset Healthcare University NHS Foundation Trust was the largest eligible organisation to fail to provide MHA data in 2021/22 [50, Appendix 2, Table 9]. For this reason, Dorset CCG was excluded from our analysis. Other eligible organisations reported fewer MHA detentions in previous years, and so this is not expected to affect our data substantially.

Furthermore, while most of the data used was collected between 2019 and 2022, the age and sex variables were extrapolated from the 2011 Census [51]. These variables may therefore not capture the true values in the population.

Only the percentage of “non-White” population was available at CCG-level. This is an issue as different ethnicities are at differing risk of involuntary hospitalisation [14], and combining these groups may have attenuated the effect of ethnicity. Furthermore, as CCGs covered large areas, the affect of neighbourhood-level ethnic density could not be assessed. Some evidence suggests that living in neighbourhoods of higher own-ethnic density could act as a protective factor against mental illness [52]. However, the effect of neighbourhood ethnic density on involuntary hospitalisation in England is not clear [53].

### Implications

The current study adds to evidence of large variation in MHA use across England [18, 19], but suggests this variation is largely unexplained by demographic, clinical, and socioeconomic differences between areas. Future research should focus on whether differences in service provision and application of the MHA between areas of England is contributing to variation in involuntary hospitalisation rates. This would allow us to better understand and tackle rising rates of MHA use.

Our findings do point to some potential causes of rising involuntary hospitalisations for mental health care. The population of England is rising, particularly in urban areas which show higher rates of ethnic diversity, deprivation, younger population, and mental illness [18]. This, paired with inadequate provision of community and inpatient mental health services [19, 42], may be driving rising rates of involuntary hospitalisation. While many of these factors were not associated with MHA use in our adjusted analyses, their influence at the area level may be masked by complex relationships. As such, the current study suggests a need to disentangle relationships between ethnicity, urbanicity, deprivation, age, mental illness, and use of community mental health services if we are to understand and combat rising rates of involuntary detention.

Our findings suggest that areas with higher severe mental illness, higher community mental health service use, and higher male population may benefit the most from interventions to reduce involuntary hospitalisation. We found that areas with higher community mental health use showed higher MHA detentions, suggesting that current community service provision in England may not be effective in reducing involuntary hospitalisation. Therefore, more research needs to assess how services can be improved to reduce involuntary hospitalisation in England. The current literature on interventions to reduce involuntary hospitalisation has found weak to moderate success of staff training, joint decision making, and integrated care interventions [54, 55]. However, researchers may need to focus on targeting potential risk factors for detention which may, in turn, help us to understand why MHA use is rising in England. For example, if help-seeking in males with severe mental illness is targeted, and this reduces the number of males being involuntarily hospitalised, then male gender can be more confidently identified as a risk factor.

## Conclusion

The current cross-sectional, ecological study used national data sets to better understand what causes variation in involuntary hospitalisation between areas in England. Building upon previous studies, we found large variation between areas that was only partly attributable to variation in demographic, clinical, and socioeconomic variables. After adjustment, areas with higher severe mental illness, community and outpatient mental health use, and male population showed higher MHA detentions. Deprivation, age, ethnicity, and urbanicity were not strongly associated with involuntary hospitalisation in the adjusted analysis. However, this may be in part due to the complex relationships between these variables. A large amount of variance between areas remained after controlling for the variables used in the study, suggesting a need to further understand area-level differences in use of the MHA. These findings can help us better understand rising rates of MHA use in England and point to areas in need of future research.

## Electronic supplementary material

Below is the link to the electronic supplementary material.

Supplementary Material 1

## Data Availability

Data was taken from the Mental Health Services Data Set Mental Health Act Statistics, Annual Figures - 2020-21(https://digital.nhs.uk/data-and-information/publications/statistical/mental-health-act-statistics-annual-figures/2020-21-annual-figures), Public Health England’s Fingertips Profiles (https://fingertips.phe.org.uk/), the Office for National Statistics Rural-Urban Classifications (https://www.gov.uk/government/statistics/2011-rural-urban-classification-lookup-tables-for-all-geographies), 2021 Census ethnic groups for Sub Integrated Care Boards (https://www.ons.gov.uk/datasets/TS021/editions/2021/versions/3), population estimates (age and sex) for CCGs (https://www.ons.gov.uk/peoplepopulationandcommunity/populationandmigration/populationestimates/datasets/clinicalcommissioninggroupmidyearpopulationestimates), and Open Geography Poral boundaries for CCGs and NHS England regions (https://geoportal.statistics.gov.uk/).

## Abbreviations

MHA: Mental Health Act 1983
CCG: Clinical Commissioning Group. These were NHS bodies responsible for delivering healthcare to local areas
BAME: Black, Asian, and Minority Ethnic
NHS: National Health Service
GP: General Practitioner
