# Supplementary material for: Area-level factors associated with variation in involuntary psychiatric hospitalisation across England: a cross-sectional, ecological study
Source: Soc Psychiatry Psychiatr Epidemiol. 2024 Aug 27;60(9):2049–59. doi: 10.1007/s00127-024-02748-5 (PMC12378475; doi:10.1007/s00127-024-02748-5)
Supplement: Supplementary file 1 — Supplementary Material 1 [file 127_2024_2748_MOESM1_ESM.docx]

**SUPPLEMENTARY MATERIAL**

**Area-Level Factors Associated with Variation in Involuntary Psychiatric Hospitalisation Across England: A Cross-Sectional, Ecological Study**

Matilda Minchin^1^ (ORCID: 0000-0002-4469-170X), Colette Christiansen^2^ (ORCID: 0000-0001-6034-5746), Lucy Maconick^1, 3^ (ORCID: 0000-0002-0174-367X), Sonia Johnson^1, 3^ (ORCID: 0000-0002-2219-1384)

**Affiliations**

1 Division of Psychiatry, University College London, Maple House, 149 Tottenham Court Road, London, W1T 7BN, UK

2 The Open University, Walton Hall, Milton Keynes, MK7 6AA, UK

3 Camden and Islington NHS Foundation Trust, St Pancras Hospital, 4 St Pancras Way, London, NW1 0PE

**Corresponding Author:** Dr Lucy Maconick, l.maconick@ucl.ac.uk.

**Contents**

[**Search Strategy** 2](#_Toc160899675)

[**Model Selection** 3](#_Toc160899676)

[**Fixed-Effects Negative Binomial Regression** 4](#_Toc160899677)

[**Mixed-Effects Sensitivity Analyses** 5](#_Toc160899678)

[**References** 6](#_Toc160899679)

# **Search Strategy**

A search was undertaken for relevant variables in Public Health England’s Fingertips Profiles [1], a collection of public health data published at different geographic levels, including CCGs. The original source of each variable was checked to ensure the most recent and complete data was extracted. In some cases data was taken from the original source, such as the Office for National statistics [2] or the NHS Digital Quality and Outcomes Framework [3]. Data was only extracted if evidenced by previous research to be associated with involuntary hospitalisations. The most recent data available at CCG-level was extracted.

The percentage of people on GP registers with recorded severe mental illness or depression was taken from the NHS Digital Quality and Outcomes Framework [3]. Data on deprivation, percentage of non-White ethnicity, and the number of community and outpatient mental health visits per 100,000 population was taken from Public Health England’s Fingertips Profiles [1]. Public Health England only reported ethnicity data at CCG-level for the broad category of non-White [1]. This included all people who did not report their ethnicity as “White UK” on the 2011 Office for National Statistics Census. Data on ethnicity breakdowns was not available at CCG-level. Office for National Statistics data was used to calculate the percentage of the CCG population who were aged 18 to 35 and who reported their sex as “male” [4]. Males and those aged 18 to 35 were chosen as these groups are at high risk of involuntary hospitalisation in England [5, 6]. Urbanicity, based on the 2011 Rural-Urban Classifications, was also taken from the Office for National Statistics [2]. This consisted of three categories: “Predominantly Urban”, “Urban with Significant Rural”, and “Predominantly Rural”. These explanatory variables had complete data for all 106 CCGs.

# **Model Selection**

Firstly, null models containing the outcome only were run with and without the random effect of NHS England region. Both the Akaike Information Criterion (AIC) and the Bayesian Information Criterion (BIC) indicated a better fit of the null model with the random effect (Null model without random effect AIC = 1272.36, BIC = 1277.67; Null model with random effect AIC = 1251.91, BIC = 1259.87). All explanatory variables that were associated with the rate of MHA detentions to p < .05 were added to a multivariate mixed-effects model. This final model had a better fit than the null model (Null model with random effect AIC = 1251.91, BIC = 1259.87; Multivariate mixed-effects model AIC = 1152.92, BIC = 1179.46). The multivariate mixed-effects model and the multivariate fixed-effects model had the same fit (Multivariate mixed-effects model AIC = 1152.92, BIC = 1179.46; Multivariate fixed-effects model AIC = 1152.92, BIC = 1179.46). As CCGs were grouped in NHS England regions, the mixed-effects model was retained.

# **Fixed-Effects Negative Binomial Regression**

Table 4: Fixed-Effects Negative Binomial Regression for the Rate of Mental Health Act Detention Across Clinical Commissioning Groups in England

|  | | Univariate Analysis | | | Multivariate Analysis | | |
| --- | --- | --- | --- | --- | --- | --- | --- |
| Variable | | IRR | 95% CI | p value | IRR | 95% CI | p value |
| Severe Mental Illness | | 3.569 | 2.793 to 4.561 | **<.001** | 2.327 | 1.803 to 3.004 | **<.001** |
| Depression | | 1.008 | .985 to 1.031 | .511 | - | - | **-** |
| Index of Multiple Deprivation | | 1.024 | 1.018 to 1.030 | **<.001** | 1.000 | .994 to 1.007 | .883 |
| % Non-White | | 1.012 | 1.007 to 1.017 | **<.001** | 1.003 | .999 to 1.007 | .097 |
| % Aged 18 to 35 Years | | 1.038 | 1.026 to 1.050 | **<.001** | 1.001 | .990 to 1.013 | .820 |
| % Male Sex | | 1.179 | 1.090 to 1.276 | **<.001** | 1.085 | 1.015 to 1.160 | **.016** |
| Community and Outpatient Mental Health Visits per 100 | | 1.020 | 1.014 to 1.026 | **<.001** | 1.013 | 1.008 to 1.017 | **<.001** |
| Urbanicity | |  |  |  |  |  |  |
| Predominantly Rural^†^ | - | - | **-** | - | - | **-** |  |
| Urban with Significant Rural | .915 | .779 to 1.073 | .273 | .962 | .870 to 1.065 | .445 |  |
| Predominantly Urban | 1.315 | 1.155 to 1.497 | **<.001** | 1.052 | .956 to 1.157 | .301 |  |

Note: Bold indicates significance to p < .05.

^†^ Reference category.

# **Mixed-Effects Sensitivity Analyses**

Table 2: Mixed Effects Negative Binomial Regression for the Rate of Mental Health Act Detention - Sensitivity Analysis for 2021 Non-White Ethnicity

|  | | Univariate Analysis | | | Multivariate Analysis | | |
| --- | --- | --- | --- | --- | --- | --- | --- |
| Variable | | IRR | 95% CI | p value | IRR | 95% CI | p value |
| Sensitivity: 2021 % Non-White | | 1.008 | 1.004 to 1.012 | **<.001** | 1.002 | .999 to 1.005 | .117 |
| Severe Mental Illness | | 3.226 | 2.452 to 4.245 | **<.001** | 2.323 | 1.795 to 3.006 | **<.001** |
| Depression | | 1.002 | .976 to 1.028 | .896 | - | - | - |
| Index of Multiple Deprivation | | 1.023 | 1.017 to 1.029 | **<.001** | 1.001 | .994 to 1.008 | .764 |
| % Aged 18 to 35 Years | | 1.032 | 1.022 to 1.043 | **<.001** | 1.001 | .989 to 1.013 | .856 |
| % Male Sex | | 1.172 | 1.092 to 1.258 | **<.001** | 1.085 | 1.015 to 1.160 | **.017** |
| Community and Outpatient Mental Health Visits per 100 | | 1.019 | 1.013 to 1.025 | **<.001** | 1.013 | 1.008 to 1.017 | **<.001** |
| Urbanicity | |  |  |  |  |  |  |
| Predominantly Rural^†^ | - | - | **-** | - | - | **-** |  |
| Urban with Significant Rural | .930 | .800 to 1.081 | .342 | .961 | .868 to 1.064 | .442 |  |
| Predominantly Urban | 1.213 | 1.067 to 1.378 | **.003** | 1.050 | .953 to 1.157 | .321 |  |

Note: Bold indicates significance to p < .05.

^†^ Reference category.

# **References**

1. Office for Health Improvement & Disparities (2023) Public Health Profiles. In: https://fingertips.phe.org.uk © Crown copyright [2023]

2. Office for National Statistics (2021) 2011 Rural Urban Classification lookup tables for all geographies. In: https://www.gov.uk/government/statistics/2011-rural-urban-classification-lookup-tables-for-all-geographies

3. NHS Digital (2021) Quality and Outcomes Framework, 2020-21. In: https://digital.nhs.uk/data-and-information/publications/statistical/quality-and-outcomes-framework-achievement-prevalence-and-exceptions-data/2020-21

4. Office for National Statistics (2020) Clinical commissioning group population estimates (National Statistics) - Ons.gov.uk. In: https://www.ons.gov.uk/peoplepopulationandcommunity/populationandmigration/populationestimates/datasets/clinicalcommissioninggroupmidyearpopulationestimates

5. Weich S, McBride O, Twigg L, et al (2017) Variation in compulsory psychiatric inpatient admission in England: a cross-classified, multilevel analysis. Lancet Psychiatry 4:. https://doi.org/10.1016/S2215-0366(17)30207-9

6. NHS Digital (2022) Mental Health Act Statistics, Annual Figures. NHS Digital. In: https://digital.nhs.uk/data-and-information/publications/statistical/mental-health-act-statistics-annual-figures
